# Molecular Imaging of Angiogenesis and Vascular Remodeling in Cardiovascular Pathology

**DOI:** 10.3390/jcm5060057

**Published:** 2016-06-06

**Authors:** Reza Golestani, Jae-Joon Jung, Mehran M. Sadeghi

**Affiliations:** 1 Cardiovascular Molecular Imaging Laboratory, Section of Cardiovascular Medicine and Yale Cardiovascular Research Center, Yale School of Medicine, New Haven, CT 06511, USA; reza.golestani@yale.edu (R.G.); jaejoon.jung@yale.edu (J.-J.J.); 2 Veterans Affairs Connecticut Healthcare System, West Haven, CT 06516, USA

**Keywords:** molecular imaging, angiogenesis, vascular remodeling, matrix metalloproteinase, αvβ3 integrin, vascular endothelial growth factor, inflammation

## Abstract

Angiogenesis and vascular remodeling are involved in a wide array of cardiovascular diseases, from myocardial ischemia and peripheral arterial disease, to atherosclerosis and aortic aneurysm. Molecular imaging techniques to detect and quantify key molecular and cellular players in angiogenesis and vascular remodeling (e.g., vascular endothelial growth factor and its receptors, αvβ3 integrin, and matrix metalloproteinases) can advance vascular biology research and serve as clinical tools for early diagnosis, risk stratification, and selection of patients who would benefit most from therapeutic interventions. To target these key mediators, a number of molecular imaging techniques have been developed and evaluated in animal models of angiogenesis and vascular remodeling. This review of the state of the art molecular imaging of angiogenesis and vascular (and valvular) remodeling, will focus mostly on nuclear imaging techniques (positron emission tomography and single photon emission tomography) that offer high potential for clinical translation.

## 1. Introduction

Angiogenesis and arteriogenesis, the processes of new capillary formation from pre-existing blood vessels, and maturation and possibly *de novo* growth of larger blood vessels are not only key components of the homeostatic response to ischemia [1], but also contribute to the development of a diverse group of pathologies, from tumor growth to atherosclerotic plaque vulnerability [2]. Similarly, vascular remodeling, persistent alterations in the structure and composition of blood vessels, is an integral component of the compensatory or pathological response of adult blood vessels to injury or hemodynamic changes [3]. Accordingly, there is considerable interest in non-invasive detection of these processes *in vivo*, both as a companion to vascular biology research and as a clinical tool for diagnosis and risk assessment, as well as tracking the effect of therapeutic interventions in patients. Traditional imaging techniques (e.g., angiography using computed tomography (CT) or magnetic resonance imaging (MRI), perfusion imaging using laser Doppler or nuclear imaging) which focus on the structure (anatomy) and function (physiology) provide important information regarding the architecture of blood vessels and tissue perfusion. However, these techniques in general provide little, if any, information regarding the *process* of neovascularization or vascular remodeling. This is in contrast to molecular imaging, which has as aim the detection, characterization, and quantification of pathobiological processes at molecular and cellular levels in living systems [4].

## 2. Imaging Technology

Various imaging modalities may be used for molecular imaging of angiogenesis and vascular remodeling [5]. Optical imaging (e.g., fluorescence and bioluminescence imaging) is versatile, relatively inexpensive, highly sensitive (picomolar to femtomolar target concentrations) and capable of real-time and repeated imaging. In addition, a large number of contrast agents (e.g., those targeting cathepsins [6,7]) are available for imaging various components of angiogenesis and vascular remodeling. However, due to limited depth of penetration of light, applications of optical imaging are restricted to small animals and invasive procedures in larger animals and humans. Furthermore, because of light attenuation and scatter, quantitative analysis of the fluorescence signal *in vivo* can be challenging. MRI can be used for molecular imaging in conjunction with targeted contrast agents, often nanoparticles loaded with gadolinium (e.g., Gd-containing liposomes targeting αvβ3 integrin [8]) or iron oxide particles. This modality has an excellent soft tissue contrast, high spatial resolution (~1 mm clinical, 25–100 μm preclinical) and unlimited depth of penetration. However, MRI has a low sensitivity and requires large amounts of contrast agents, which can be toxic or interfere with the biological system. Given its reliance on nanoparticles, targeted MRI is most suitable for endothelial targets, as well as macrophage imaging. Similar to MRI, CT is a high resolution imaging modality which can be combined with contrast-loaded nanoparticles for molecular imaging. However, the low sensitivity of CT is a major practical limitation and therefore, CT is often used as companion to nuclear or optical techniques for anatomic localization. Ultrasound is a low-cost imaging modality which can be used in combination with functionalized, gas-filled microbubbles for molecular imaging (e.g., to target αvβ3 integrin [9]). Versatility, relatively high spatial and temporal resolution and high sensitivity are some of the strengths of ultrasound-based imaging. However, given its reliance on microbubbles, applications of ultrasound-based imaging are limited to intravascular targets. Nuclear imaging techniques (positron emission tomography (PET) and single photon emission tomography (SPECT)) detect the biodistribution of a radiolabeled tracer (often in the nanomolar amounts) within the body. Both techniques are highly sensitive (targets at 10^−10^ to 10^−12^ M concentration), highly quantitative and not limited by depth of penetration. However, both techniques suffer from a relatively poor spatial resolution (5–10 mm clinical, ~1 mm preclinical), which mandates hybrid imaging with high resolution CT or MRI imaging for anatomical localization. A PET radioisotope (e.g., F-18, Cu-64) releases positrons from its nucleus. The collision of a positron with an electron releases two photons traveling at opposite directions which can be detected by a PET scanner. Radioisotopes used for SPECT (e.g., Tc-99m) release gamma rays, which are detected with gamma cameras. Importantly, SPECT is less sensitive than PET. However, unlike PET photons which have an energy of 511 Kev, SPECT gamma rays have different energies, enabling simultaneous imaging of multiple radioisotopes [5].

## 3. Role of Molecular Imaging

Angiogenesis (and neovascularization in general) plays an important role in a broad range of cardiovascular pathology. As the promise of therapeutic neovascularization to treat symptoms in myocardial ischemia and peripheral arterial disease in humans remains unfulfilled, there is considerable interest in molecular imaging for assessing angiogenesis *in vivo*. Through quantitative assessment of key intermediaries in longitudinal studies these techniques can help elucidate the biological basis of therapeutic effectiveness (or failure); thus advance pathobiology research and help optimize therapeutic interventions. In parallel, angiogenesis plays a deleterious role in atherosclerosis as the presence of extensive neovessels (and intra-plaque hemorrhage) is linked to atherosclerotic plaque vulnerability [10]. Accordingly, targeting the neovasculature in the plaque for imaging may be useful for risk stratification of patients with coronary and carotid artery disease. At the same time, these neovessels provide a venue to deliver a treatment specifically to the plaque. Given the many applications of pro- and anti-angiogenic therapy, molecular imaging of the processes involved in angiogenesis (and neovascularization in general) could help identify those patients who would benefit most from pro- or anti-angiogenic therapy, monitor the effect of treatment, and advance drug discovery research.

Vascular remodeling is a key feature of most vascular diseases. The two components of remodeling, changes in vessel wall thickness (e.g., neointima formation) and size (e.g., positive remodeling) are important in varying degrees in various vascular pathologies. As such, while neointima formation is a hallmark of the response to mechanical injury, expansive remodeling is the key feature in aortic aneurysm. Vascular remodeling may be focal, as in aneurysm, or diffuse as in pulmonary arterial hypertension and transplant vasculopathy. In atherosclerosis, expansive remodeling initially contributes to maintaining the luminal area to preserve blood flow. However, this also leads to increased risk of plaque rupture which may result in acute coronary syndrome and death. Accordingly, imaging components of vascular remodeling may not only help elucidate the pathobiological process, but also may be useful for early diagnosis, risk stratification and tracking the effect of therapeutic interventions in vascular pathologies. Ultimately, obtaining a full picture of the remodeling process requires the combination of molecular imaging with structural imaging (e.g., with CT or MRI).

There is a large body of literature on preclinical (and in some cases, clinical) evaluation of imaging targets for detection of key molecular players involved in angiogenesis and vascular remodeling. Some of these, VEGF (vascular endothelial growth factor) pathway, αvβ3 integrin and protease activation, are reviewed in the following sections. However, it is important to note that a surrogate marker not directly involved in the pathogenic pathway, could potentially be as effective as a molecular mediator as target for molecular imaging.

### 3.1. Imaging of VEGF Pathway

VEGF is a major proangiogenic growth factor [11]. As such, molecular imaging of the components of VEGF signaling pathway may provide important information regarding the angiogenic process. The effects of VEGF *in vivo* are mediated by tyrosine kinase VEGF receptors [VEGFRs]) and their co-receptors [2]. There are three VEGFRs (VEGFR-1, -2, and -3). Of these, VEGFR-2 is the key receptor for many proangiogenic effects of VEGF in endothelial cells (ECs). VEGF-A, the main pro-angiogenic isoform of the family, primarily interacts with VEGFR-1 and VEGFR-2. Both VEGF and its receptors are upregulated in response to ischemia and accordingly are potential targets for molecular imaging of angiogenesis. In addition, VEGFRs are expressed in inflammatory cells and may serve as targets for imaging in inflammatory states [12,15]. *In vivo* imaging of VEGFRs may be achieved using radiolabeled VEGF_121_, a VEGF isoform that lacks the heparin binding domain of VEGF_165_ [12]. Accordingly, ^64^Cu-DOTA-VEGF_121_ PET was used to track VEGFR expression in the myocardium after myocardial infarction in a rat study [13]. In this study, ^64^Cu-DOTA-VEGF_121_ uptake was detected in the infarct region and peaked on day 3 post-infarct. A parallel increase in VEGFR-1 and -2 expression in the infarct area suggested that the tracer is indeed targeting VEGFRs. The specificity of the cardiac signal was demonstrated using a mutant VEGF_121_ that does not bind to VEGFRs. The same tracer was used in a murine model of ischemia-induced hindlimb angiogenesis, where the highest uptake in the ischemic hindlimb was detected on postoperative day 8, when VEGFR-2 expression was maximal. Interestingly, exercise increased ^64^Cu-DOTA-VEGF_121_ uptake in conjunction with increased microvessel density and VEGFR-2 expression, supporting the value of this tracer for non-invasive tracking of dynamic changes in angiogenesis [14]. VEGFRs’ expression in inflammatory cells [12,15], and VEGF_121_ binding to neuropilins potentially complicates the interpretation of these studies [16].

In vascular remodeling, VEGF plays a complex role [15,17,18]. The feasibility of VEGF imaging of vascular remodeling was shown in a chimeric human-mouse model of transplant vasculopathy in which segments of human coronary artery were transplanted to severe combined immunodeficiency mice. Reconstitution of the animals with allogeneic human peripheral blood mononuclear cells led to significant expansion of the total vessel and neointima areas over a period of 4 weeks. *Ex vivo* near infrared fluorescence imaging following *in vivo* administration of Cy5.5-labeled single-chain VEGF_121_ showed significant focal uptake of the tracer in coronary artery transplants at 4 weeks [19]. This uptake was mostly in the neointima and correlated with VEGFR-1, but not VEGFR-2 expression, suggesting a potential role for VEGF-based imaging in vascular remodeling.

Bevacizumab, a humanized monoclonal antibody against VEGF-A is used in the clinic for cancer treatment. Accordingly, *in vivo* visualization of VEGF-A in tumor/tissue may help determine which patient would benefit from anti-VEGF therapy. To this end, bevacizumab has been radiolabeled and used to assess VEGF-A expression in human tumors *in vivo* [20,21]. To date, this agent has not been used for cardiovascular imaging *in vivo*. In a feasibility study, ^89^Zr-Bevacizumab was evaluated for *ex vivo* imaging of excised carotid artery atherosclerotic plaques [22]. Using high-resolution microPET imaging, a specific pattern of ^89^Zr-bevacizumab accumulation was noted which showed an excellent correlation with VEGF immunohistochemical staining scores. This study also showed that the areas of high VEGF staining correspond to areas of high CD68 staining within carotid endarterectomy specimens, suggesting a role for VEGF-targeted imaging to assess inflammation in atherosclerosis. Because bevacizumab is a humanized monoclonal antibody further validation of the tracer requires studies on human tissues before its translation to clinical imaging.

### 3.2. Imaging of αvβ3 Integrin

Integrins are a group of heterodimeric receptors that regulate cell migration, and cell-cell and cell-extracellular matrix protein interactions in angiogenesis [23,24]. αvβ3 integrin is highly expressed on activated endothelial cells (while it is only modestly expressed on normal blood vessels) [23,25]. Accordingly, αvβ3 integrin expression is strongly associated with angiogenesis in tumors and cardiovascular pathology and can thus be used as a target for depiction of angiogenesis through molecular imaging techniques. The role of αvβ3 integrin in angiogenesis remains to be fully defined. The interruption of proangiogenic pathways by αvβ3 integrin function blocking agents, enhanced angiogenesis in β3 knockout mice, and recent failure of the αvβ3 antagonist, cilengitide, in patients with glioblastoma highlight the complex nature of integrin function in angiogenesis [26,27].

αvβ3 integrin interacts with several extracellular matrix proteins (including fibronectin and vitronectin) through a tripeptide sequence, arginine-glycine-aspartate (RGD) [28]. As such, RGD peptides have been used extensively as imaging agents (or as their components) for imaging of αvβ3 integrin in cancer and cardiovascular diseases. In addition to αvβ3, several other integrins, including α5β1 and αvβ5 interact with RGD peptides [29]. This interaction which is modulated by RGD flanking residues and three dimensional structure should be considered in the interpretation of imaging studies involving RGD-based imaging agents. Several RGD-based tracers are currently under evaluation in clinical trials for tumor imaging [30,31]. Among these are monomeric (^18^F-galacto-RGD [32] and ^18^F-Fluciclatide [33]), and dimeric (^18^F-FPPRGD2 [34] and ^18^F-AlF-NOTA-PRGD2 [35]) RGD-based compounds for PET, and ^99m^Tc-NC100692 [36] for SPECT imaging. Many of these agents show favorable pharmacokinetic, target accumulation and binding affinities in humans.

**Figure 1:**
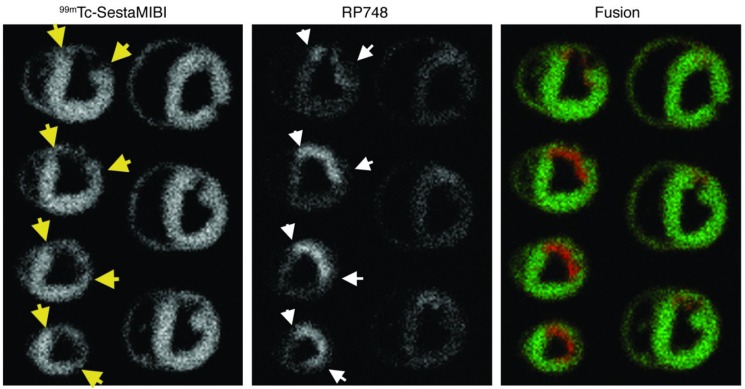
Integrin αvβ3-targeted imaging of myocardial angiogenesis. Example of *ex vivo* ^99m^Tc-sestamibi (reflecting perfusion, (**left**)) and ^111^In-RP748 (reflecting αvβ3 integrin expression and activation, (**center**)) and fused color-coded (**right**) images of canine myocardium at three weeks post myocardial infarction induced by left anterior descending coronary artery occlusion. Yellow arrows point to the margins of the perfusion defect, and white arrows show the area of increased ^111^In-RP748 uptake. On fused images perfusion tracer uptake is in green. RP748 accumulation (in red) is localized in the areas of perfusion defect. Published with permission of the American Society for Clinical Investigation, from “Noninvasive imaging of myocardial angiogenesis following experimental myocardial infarction”, Meoli, D.F., *et al. J. Clin. Invest*. **2004**, *113(12)*, 1684–1691 [38]; permission conveyed through Copyright Clearance Center, Inc.

**Figure 2:**
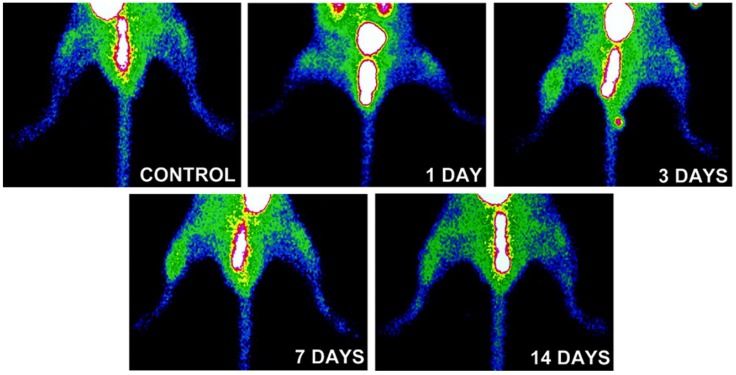
Integrin αvβ3-targeted imaging of hindlimb angiogenesis. Examples of *in vivo* αvβ3-targeted planar imaging of a control mouse (top) and mice subjected femoral artery ligation to induce hindlimb ischemia, demonstrating focal uptake of the RGD-based tracer, NC100692 is the ischemic hindlimb, maximal at 3 and 7 days after ischemia. Published with permission of Wolters Kluwer Health from “Noninvasive imaging of angiogenesis with a^99m^Tc-labeled peptide targeted at α_v_β_3_ integrin after murine hindlimb ischemia”, Hua, J., *et al. Circulation* **2005**, *111 (24)*, 3255–3260 [43].

Beyond imaging of angiogenesis in cancer, RGD imaging has been studied for evaluation of angiogenesis in rodent models of myocardial infarction and peripheral vascular disease. ^111^In-RP748 is a peptidomimetic tracer which binds with high affinity to activated αv integrins [37]. *In vivo* integrin-targeted imaging using this tracer in rat and canine models of myocardial infarction demonstrated specific uptake of the tracer in regions of decreased perfusion, where there was enhanced integrin expression and angiogenesis (Figure 1) [38]. Similarly, αv integrin-targeted PET imaging in a rat model of myocardial ischemia and reperfusion using ^18^F-Galacto-RGD as the imaging agent, showed uptake of the tracer in the infarct area starting three days after injury and peaking between 1 and 3 weeks after injury. Importantly, ^18^F-Galacto-RGD uptake correlated with CD31 staining-ascertained microvessel density in the myocardium [39]. Based on the premise that ^68^Ga tracers offer the advantage of fast production without need of cyclotrons, the same group used a ^68^Ga-labeled RGD compound and compared its *in vivo* characteristics with ^18^F-galacto-RGD [40] in a similar set of studies. In this study β3 integrin expression was higher in the infarct and border zones of rat myocardium compared to the remote area, and ^68^Ga-NODAGA-RGD and ^68^Ga-TRAP-RGD showed a high infarct-to-remote accumulation ratio in correlation with β3 integrin staining. Similar results have been reported using other RGD tracers [41,42]. The feasibility of imaging angiogenesis in peripheral arterial disease was shown in a mouse model of hindlimb ischemia. In this model, angiogenesis in the ischemic limb was monitored by *in vivo* imaging over a 14 days period using a ^99m^Tc-labeled RGD-based compound, NC100692 (Figure 2) [43]. The authors noted increased uptake of NC100692 in the ischemic limb at days 3 and 7. Furthermore, there was co-localization of a fluorescent analog of NC100692 with CD31-positive cells in the ischemic limb, whereas in the control limb there was no positive RGD staining despite abundance of CD31-positive cells. This is consistent with preferential binding of this tracer to the active form of the integrin.

RGD imaging has also been used to image vascular remodeling and characterize atherosclerotic lesions. As discussed above, angiogenesis plays an important role in atherosclerotic plaque development and vulnerability. However, given the relative abundance of macrophages in atherosclerotic plaque and αvβ3 expression on macrophages [44], in molecular imaging studies the RGD signal in atherosclerosis can represent both angiogenesis and inflammation, which are distinct, yet related processes. This complexity can be addressed, at least in part using imaging agents with intravascular retention. As such, αvβ3 integrin-targeted paramagnetic nanoparticles have been used to image neovascularization in rabbit atherosclerotic lesions by MRI [8], and to deliver drugs (e.g., the antiangiogenic agent, fumagillin) specifically to the plaque [45]. Unlike nanoparticles, nuclear–based imaging agents are often small molecules that readily cross the dysfunctional endothelial barrier in atherosclerosis. This is highlighted in a study in atherosclerotic mice, where higher ^18^F-galacto-RGD uptake was shown in aortae of LDLR^−/−^ ApoB^100/100^ mice compared to aortae of control wild-type mice [46]. Accumulation of ^18^F-galacto-RGD was also higher in areas with atherosclerotic plaques compared to adjacent normal artery and adventitial tissue and tracer uptake correlated with vessel wall macrophage content, whereas no correlation with markers of angiogenesis were noted. The association between RGD signal and plaque macrophages was further demonstrated by co-immunostaining in human carotid endarterectomy specimens using a fluorescent RGD peptide [47]. In another study, microPET was used for imaging of excised carotid endarterectomy specimens following *ex vivo* incubation with an αvβ3 integrin-targeted tracer, ^18^F-RGDk5 [48]. In this study, there was heterogeneous accumulation of the tracer in the endarterectomy specimens, which co-localized with CD31 and αvβ3 integrin in areas of higher tracer uptake. Finally, ^99m^Tc-NC100692 microSPECT/CT in a murine model of carotid aneurysm showed significantly higher uptake of the tracer in the aneurysmal artery compared to the contralateral vessel, and the uptake correlated with vessel wall inflammation [44].

### 3.3. Imaging of Protease Activation

Matrix reorganization is an integral part of tissue remodeling observed in vascular remodeling, angiogenesis and other cardiovascular pathology. Members of the cysteine protease family of cathepsins expressed by ECs, VSMCs and inflammatory cells are upregulated in vascular remodeling and may serve as targets for imaging. To date, the main applications of cathepsin imaging have been in murine models of atherosclerosis [7] and calcific aortic valve disease (CAVD) [49] using activatable probes that generate a fluorescence signal upon cleavage of a specific substrate. Both cathepsin B and K activity are shown by *ex vivo* fluorescence imaging to be upregulated in atherosclerosis in the vicinity of macrophages [6,7]. A major limitation of this technique is its reliance on activatable fluorescent probes which have limited use for non-invasive imaging in humans.

**Figure 3:**
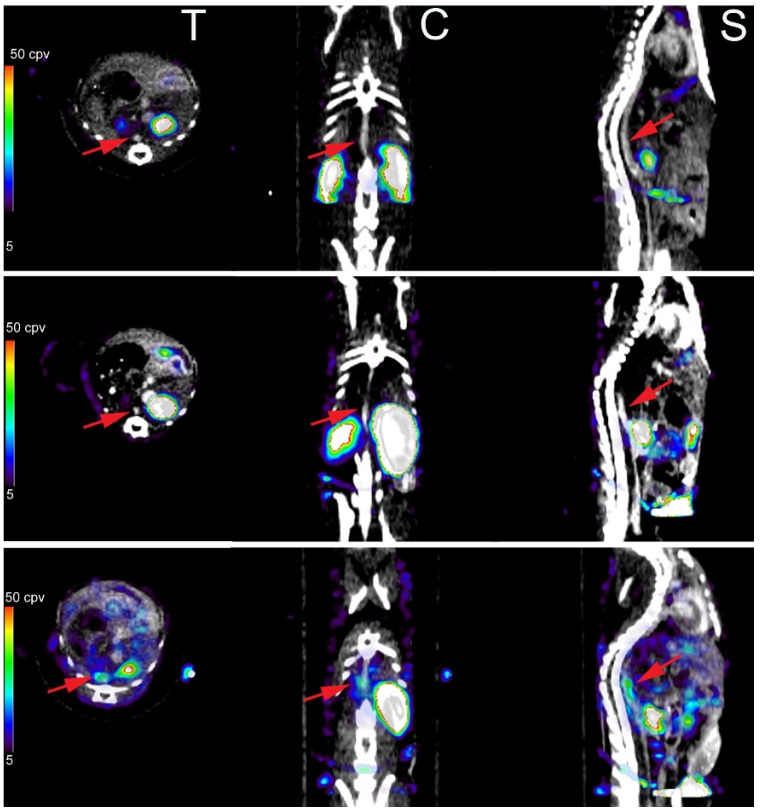
MMP-targeted imaging of vascular remodeling in abdominal aortic aneurysm. Examples of fused ^99m^Tc-P805 microSPECT and CT images of matrix metalloproteinase activation in abdominal aortae of a control mouse ((**top**) row) and animals infused with angiotensin II to induce abdominal aortic aneurysm. A mouse infused with angiotensin II for 4 weeks that developed abdominal aortic aneurysm shows RP805 uptake in the aneurysm ((**bottom**) row), whereas there is no notable tracer uptake in the abdominal aortae of the control mouse (top row) and an angiotensin II-infused mouse that did not develop aneurysm ((**middle**) row). T: transvers, C: coronal, S: sagittal. Arrows point to abdominal aortae, identified by CT angiography. Published with permission of Wolters Kluwer Health from “Imaging vessel wall biology to predict outcome in abdominal aortic aneurysm”, Golestani, R., *et al. Circ. Cardiovasc. Imaging* **2015**, *8(1)*, e002471 [56].

MMPs are a large family of endopeptidases with different, but overlapping substrate specificity. Various cells in the cardiovascular system express and activate MMP and their inhibitors, tissue inhibitors of MMPs (TIMPs), yet inflammatory cells are major sources of MMPs in many cardiovascular diseases. Given the role of MMPs in vascular remodeling, MMP imaging can provide important information regarding the remodeling process and its regulators. Both the proliferative component of vascular remodeling and changes in the vessel size depend on MMP activation. Accordingly, MMP imaging may detect the dominant process in different forms of vascular remodeling. In addition to activatable fluorescent probes [50], which suffer from the same limitations of cathepsin probes discussed above, a number of imaging agents targeting MMPs (especially their activated forms) have been developed that have shown promise in preclinical studies of cardiovascular pathology. These include P947, a gadolinium-labeled agent with modest affinity for MMPs and several other enzymes, which has been used for detection of protease activity in animal models of atherosclerosis by MRI [51,52]. Other imaging agents in preclinical studies include a family of hydroxamate-based nuclear imaging agents (including the ^111^In-labeled RP782 and ^99m^Tc-labeled RP805) that target MMP activation in general (panMMP) and have been extensively evaluated in animal models of vascular and valvular remodeling. In a murine model of wire injury to carotid arteritis, which is characterized by significant neointima formation, MMP activation detected by RP782 microSPECT/CT imaging was higher in the injured carotid compared to control, sham-operated artery, and importantly the signal paralleled weekly changes in the neointima area over a 4-week period [53]. In addition, an intervention aimed at reducing remodeling, *i.e.*, dietary intervention, led to a decrease in MMP signal [54]. Similarly, in a mouse model of carotid aneurysm (where expansive remodeling is a key feature of the disease) the development of aneurysm was associated with enhanced MMP activation and the MMP signal detected *in vivo* by RP782 microSPECT/CT imaging correlated with vessel wall inflammation [55]. In animals undergoing serial MMP imaging at 2 and 4 weeks after surgery, the MMP signal intensity at 2 weeks predicted the aneurysm size at 4 weeks, highlighting the contribution of MMPs to the remodeling process [55]. The predictive value of MMP imaging was confirmed in a murine model of angiotensin-II-induced aneurysm where there is spontaneous rupture of aortic aneurysm in a subset of animals (Figure 3) [56]. In this model RP805 microSPECT/CT imaging at 1 week after angiotensin II infusion predicted aneurysm development or rupture at 4 weeks, highlighting the contribution of molecular imaging to vascular biology research and potentially, clinical medicine [56]. The association between MMP activation detected *in vivo* and inflammation, mainly macrophage content of the vessel wall can also be seen in animal models of atherosclerosis. RP782 and RP805 imaging of MMP activation in apoE^−/−^ mice fed a high fat diet to induce atherosclerosis showed heterogeneous uptake of the tracer along the aorta, highlighting the heterogeneity of the disease in these animals [57,58]. In addition, therapeutic interventions, whether changes in the diet or lipid-lowering agents, reduced the MMP signal *in vivo* [54].

Despite the role of MMPs in angiogenesis, little work has been done to evaluate MMPs as target for imaging angiogenesis. However, another potentially promising application of MMP imaging in cardiovascular pathology is in calcific aortic valve disease (CAVD), which is the main cause of aortic stenosis. CAVD pathogenesis overlaps that of atherosclerosis. In response to stress, such as abnormal shear stress, valvular ECs are activated to overexpress adhesion molecules, such as vascular cell adhesion molecule-1 (VCAM-1) and intercellular adhesion molecule-1 (ICAM-1). This leads to inflammatory cell recruitment to the aortic valve, which triggers a process that leads to leaflet fibrosis, calcification, and ultimately, aortic stenosis [59]. A number of MMPs, including MMP-2, -3, -7, -9, -12 and -13, are up-regulated in CAVD [60,61,62,63]. The potential role of MMP imaging in CAVD was highlighted in a recent report on RP805 microSPECT/CT imaging of CAVD in high fat fed apoE^−/−^ mice, where the peak MMP signal *in vivo* preceded the development of more severe aortic valve calcification (and stenosis) [64].

### 3.4. Imaging of Other Targets

A number of imaging agents (e.g., ^18^F-fluorodeoxyglucoe, ^18^F-sodium fluoride, ultrasmall superparamagnetic particles of iron oxide) are undergoing clinical evaluation for imaging inflammation and calcification in atherosclerosis, aneurysm and CAVD by PET and MRI. While many uncertainties persist regarding the biological basis of their signals on *in vivo* images, ongoing clinical trials seek to establish the role of these advanced techniques in patient risk stratification and tracking the effects of therapeutic interventions. The reader is referred to several recent reviews by our group for in-depth discussion of these issues [65,66,67,68].

## 4. Conclusions

We have focused this review mostly on nuclear imaging techniques and commonly used targets for imaging of angiogenesis in cardiovascular pathology and vascular (and valvular) remodeling. Accordingly, we have omitted a number of other imaging targets and imaging modalities that have been evaluated more or less successfully in high quality preclinical and clinical studies. Clearly, molecular imaging is an emerging technique at the verge of clinical translation, with great promise for advancing basic biology research and addressing diagnostic gaps in the clinic. Like any new technique, advances in this area need critical evaluation to define strengths and limitations and to ascertain applicability in specific research or clinical scenarios.

## Abbreviations

The following abbreviations are used in this manuscript:

CAVD	calcific aortic valve disease
CT	computed tomography
MRI	magnetic resonance imaging
PET	positron emission tomography
SPECT	single photon emission tomography
VEGF	vascular endothelial growth factor
VEGFR	vascular endothelial growth factor receptor
RGD	arginine-glycine-aspartate
TIMP	tissue inhibitor of matrix metalloproteinases
ICAM-1	intercellular adhesion molecule-1
